# Role of cytokines in myocardial ischemia and reperfusion

**DOI:** 10.1080/09629359791668

**Published:** 1997-06

**Authors:** H. S. Sharma, D. K. Das

**Affiliations:** 1 Department of Pharmacology Erasmus University Rotterdam The Netherlands; 2 Department of Surgery University of Connecticut Health Center Farmington USA

## Abstract

Mediators of myocardial inflammation, predominantly cytokines, have for many years been implicated in the healing processes after infarction. In recent years, however, more attention has been paid to the possibility that the inflammation may result in deleterious complications for myocardial infarction. The proinflammatory cytokines may mediate myocardial dysfunction associated with myocardial infarction, severe congestive heart failure, and sepsis. A growing body of literature suggests that inflammatory mediators could play a crucial role in ischemia–reperfusion injury. Furthermore, ischemia–reperfusion not only results in the local transcriptional and translational upregulation of cytokines but also leads to tissue infiltration by inflammatory cells. These inflammatory cells are a ready source of a variety of cytokines which could be lethal for the cardiomyocytes. At the cellular level it has been shown that hypoxia causes a series of well documented changes in cardiomyocytes that includes loss of contractility, changes in lipid metabolism and subsequent irreversible cell membrane damage leading to cell death. For instance, hypoxic cardiomyocytes produce interleukin-6 (IL-6) which could contribute to the myocardial dysfunction observed in ischemia reperfusion injury. Ischemia followed by reperfusion induces a number of other multi-potent cytokines, such as IL-1, IL-8, tumor necrosis factor-α (TNF-α), transforming growth factor-β1 (TGF-β1) as well as an angiogenic cytokine/ growth factor, vascular endothelial growth factor (VEGF), in the heart. Intrestingly, these multipotent cytokines (e.g. TNF-α) may induce an adaptive cytoprotective response in the reperfused myocardium. In this review, we have included a number of cytokines that may contribute to ventricular dysfunction and/or to the cytoprotective and adaptive changes in the reperfused heart.


Mediators of Inflammation, **6**, 175–183 (1997)

### Role of cytokines in myocardial ischemia and reperfusion

H. S. Sharma[1,CA] and D. K. Das[2]

[1]Department of Pharmacology, Erasmus University, Rotterdam, The Netherlands [2]Department of Surgery, University of Connecticut Health Center, Farmington, USA

[CA]Corresponding Author
Tel: (+31) 10 4087963
Fax: (+31) 10 4366839

**Mediators of myocardial inflammation, predominantly cytokines, have for many years been implicated in the healing processes after infarction. In recent years, however, more attention has been paid to the possibility that the inflammation may result in deleterious complications for myocardial infarction. The proinflammatory cytokines may mediate myocardial dysfunction associated with myocardial infarction, severe congestive heart failure, and sepsis. A growing body of literature suggests that inflammatory mediators could play a crucial role in ischemia–reperfusion injury. Furthermore, ischemia–reperfusion not only results in the local transcriptional and translational upregulation of cytokines but also leads to tissue infiltration by inflammatory cells. These inflammatory cells are a ready source of a variety of cytokines which could be lethal for the cardiomyocytes. At the cellular level it has been shown that hypoxia causes a series of well documented changes in cardiomyocytes that includes loss of contractility, changes in lipid metabolism and subsequent irreversible cell membrane damage leading to cell death. For instance, hypoxic cardiomyocytes produce interleukin-6 (IL-6) which could contribute to the myocardial dysfunction observed in ischemia–reperfusion injury. Ischemia followed by reperfusion induces a number of other multi-potent cytokines, such as IL-1, IL-8, tumor necrosis factor-α (TNF-α), transforming growth factor-β1 (TGF-β1) as well as an angiogenic cytokine/growth factor, vascular endothelial growth factor (VEGF), in the heart. Intrestingly, these multipotent cytokines (e.g. TNF-α) may induce an adaptive cytoprotective response in the reperfused myocardium. In this review, we have included a number of cytokines that may contribute to ventricular dysfunction and/or to the cytoprotective and adaptive changes in the reperfused heart.**

**Key words:** Heart, ischemia–reperfusion, cytokine, TNF-α, IL-1, IL-6, TGF-β

### Myocardial Ischemia and Reperfusion

Coronary artery disease is one of the most common cardiac disease and it takes a heavy toll of lives in industrialized countries. Stenosis of a coronary artery leads to the myocardial ischemia which provokes tissue injury that could be reversible or irreversible depending on the severity and duration of myocardial ischemia. In the case of reversible tissue injury, structural and functional properties of the myocardium completely recover after reperfusion, whereas after irreversible injury, tissue does not recover upon reperfusion and the myocardium becomes necrotic.[1,2] The occurrence of myocardial infarction during an acute coronary occlusion indicates not only the lack of oxygen and blood supply but also the disruption of defense and adaptive mechanisms of the myocardium against ischemia. Myocardial responses to a critical coronary stenosis in terms of adaptation and defense could be (1) long lasting (hours to days) contractile dysfunction in the absence of any cell death (stunned myocardium);[3,4] (2) tolerance towards ischemia after a short period of supply–demand mismatch (preconditioned myocardium);[5,6] (3) myocardial angiogenesis i.e.

© 1997 Rapid Science Publishers growth of blood vessels and development of collateral circulation;[7,8] and finally, (4) atrophy of cardiac myocytes in chronic coronary artery disease.[9]

### Molecular Phenotype of Reperfused Myocardium

Myocardial ischemia can induce an adaptive and angiogenic process which consists of a number of sequential events including endothelial cell proliferation, migration and tissue infiltration; these processes are probably regulated by polypeptide growth factors and cytokines. Recently, we and others have reported that brief periods of ischemia leave myocardium stunned without cellular necrosis.[3,4,10] Stunning swine myocardium with two cycles of a 10 min left anterior descending coronary artery (LAD) occlusion followed by reperfusion increases tolerance to a subsequent longer period (60 min) of ischemia.[6] To get an insight into the myocardial molecular response in ischemic reperfused swine myocardium, we studied the expression pattern of a number of genes which could play a pivotal role in cytoprotection and adaptation in the heart. These were the proto-oncogenes (c-*myc*, c-*fos*, c-*jun*, and *egr*-1),[11] heat shock proteins (HSPs; HSP-70, HSP-27, HSP-32, HSP-8),[6,9,12] calcium regulatory proteins[13,14] and angiogenic growth factors.[7,15,16] Proto-oncogene products have been implicated in almost every aspect of growth control, from the binding of growth factors to cell surface receptors, to signal transduction and the regulation of transcription.[11,17,18] HSPs are a highly conserved group of cellular proteins which are expressed in almost every type of organism from bacteria to man.[19,20] HSP-70 binds to ATP and helps in translocating cytoplasmic proteins in the cells and HSP-27 migrates to the nucleus in response to stress, acts as molecular chaperone and plays an important role in signal transduction.[19,21–25] A number of groups, including ours, have reported that myocardial ischemia and reperfusion induce HSP-70 and HSP-27.[6,9,21,25] Brief periods of myocardial ischemia in rabbits also cause a rapid expression of HSP-70, which is detectable at the protein level within 2 hours.[26] Physiological relevance of such induced HSPs is believed to provide myocardial cytoprotection and adaptation after an ischemic episode.[20,23,25,27,28] Ubiquitin, another highly conserved small HSP was found to be up-regulated in the reperfused myocardium.[10,29] It is believed that enhanced expression of ubiquitin in response to stress is followed by its reversible conjugation with abnormal proteins destined for degradation.[29–31] We believe that brief coronary occlusions–reperfusions would cause molecular damage in cardiac myocytes and such damage requires the involvement of HSPs in rescuing several vital proteins and restoring myocardial function. In contrast, irreversible myocardial damage requires rapid disposal via the ubiquitin system (ubiquitination and subsequent proteolysis) to ensure proper functioning of the surviving cells. Anti-oxidative enzyme systems (such as catalase and $Mn^{2+}$ SOD) have also been implicated in the adaptive mechanisms underlying myocardial ischemia–reperfusion.[32] Very recently, we have shown that myocardial ischemia and reperfusion lead to the induction of heme oxygenase-1 (also known as HSP-32), an enzyme that generates antioxidant biliverdin; the stimulus for its en- hanced expression appears to be oxygen free radicals.[12,33]

### Expression of Cytokines in the Heart

### Transforming growth factor-β1

Transforming growth factor-β1 (TGF-β1) is a 25 kDa homodimeric polypeptide differentiation factor found in platelets and most organs, including the heart.[34–36] By using reverse transcriptase–polymerase chain reaction (RT–PCR) and Northern blot analysis, we have shown that chronic myocardial ischemia resulted in enhanced expression of TGF-β1 mRNA.[15] *In situ* hybridization studies revealed that TGF-β1 specific mRNA transcripts were predominantly localized in cardiac myocytes near fibrotic tissue and not in the area of the inflammatory infiltrate.[36] Furthermore, immunoblot analysis with a polyclonal anti-TGF-β1 antibody showed a specific band of 25 kDa in myocardial protein extracts from normal and chronically ischemic hearts.[36] Immunoreactive TGF-β1 was localized in the cardiomyocytes. Purkinje cells of the conduction system were consistently stained with TGF-β1 specific antibodies[15,36] indicating that TGF-β1 may influence the degree of differentiation in these cells. The cellular source of TGF-β1 in the heart is controversial. Thompson *et al.*[34] found TGF-β1 mRNA and protein in the cardiac myocytes of the infarcted tissue, whereas Eghbali[35] demonstrated mRNA expression of TGF-β1 only in the nonmyocyte fraction of cardiac tissue.

Although TGF-β1 is an inhibitor of endothelial cell proliferation it has been found to be angiogenic when injected subcutaneously into newborn mice[37] or when applied locally in wound healing experiments.[38] The angiogenic response to TGF-β1 application appears to be an indirect one, the primary response is the chemoattraction for monocytes which then, in turn, stimulate angiogenesis.[37,39] TGF-β1 has been shown to contribute to myocardial protection during ischemic injury;[40] a single bolus dose of TGF-β1 to a rat markedly protected the heart against reperfusion injury as assessed by loss of myocardial creatinine kinase activity and a reduction in circulating TNF-α levels.[41] Studies suggest that TGF-β1 might be an important molecule in cardiac embryogenesis, hypertrophy, atherogenesis, healing of myocardial infarction and in the development of coronary collaterals.[34,36,39,41,42] TGF-β1 also maintains the contractility of cultured cardiomyocytes and blocks the supressive effects of IL-1 on their beating rate by down-regulating the expression of NO-synthase.[43] Thus, TGF-β1 could be a clinically important cytokine in the treatment of reperfusion injury and inflammatory disease in the heart.

### Tumor necrosis factor-α

Tumor necrosis factor-α (TNF-α) is a 17 kDa multipotent cytokine produced mainly by activated macrophages that has been implicated in several biologic processes including inflammation, immunoregulation, and angiogenesis.[44] It acts directly on vascular endothelium as well as on cardiomyocytes to increase the adhesion of leukocytes during inflammation.[45,46] TNF-α is similar in many ways to TGF-β as both polypeptides induce angiogenesis *in vivo*, promote tube formation *in vitro*, but inhibit endothelial cell proliferation; this indicates that TNF-α is an indirect angiogenic growth factor which may stimulate other cells to produce angiogenic factors such as VEGF.[45–47] Biological effects of TNF-α in the target cell (cardiomyocytes) are initiated by its binding to high affinity cell surface receptors.[48–50] TNF receptors channel signals to cytoplasm and nucleus, and thereby initiate profound alterations in the metabolic pathway and nuclear transcription.[51] By using RT–PCR, we detected mRNAs encoding TNF-α in the porcine normal as well as ischemic myocardium.[15] By Northern hybridization, we observed that TNF-α specific mRNA expression was induced in the ischemic myocardium as compared with the level found in normally perfused myocardium.[15] Expression of TNF-α and its receptors has been shown in failing human hearts indicating a pivotal role for this cytokine in pathogenesis.[52,53] Furthermore, the literature suggests that TNF-α mRNA is transcribed in the adult heart at a detectable level and may play a role in the inflammatory processes caused by myocardial ischemia.

### TNF-α induced cytoprotective mechanisms in the heart

Dysfunction of the coronary endothelium as well as injury to cardiac muscle cells are the consequences of myocardial ischemia and reperfusion. Among many factors, TNF-α is also released into the postischemic myocardium.[41,54,55] These ischemia-induced cytokines may mimic local cellular injury and may contribute to the changing molecular phenotype of the postischemic myocardium. An increase in both local expression and circulating TNF-α has been reported in experimental animals and in patients with myocardial ischemia and infarction.[54,55] Interestingly, pretreatment of rat hearts with TNF-α was found to be protective against ischemia and reperfusion injury.[56] However, in the normal heart, TNF-α may exert negative inotropic effects by directly altering intracellular calcium homeostasis in a concentration- and time-dependent manner.[57] Many studies have demonstrated that TNF-α induces phosphorylation of a stress protein of about 28 kDa in a number of cell types including cardiomyocytes[58–60] and this phosphorylation results from stimulation of a G protein-coupled signal transduction pathway involving the mitogen activated protein (MAP) kinase.[61–62]

Perfusion of spontaneously contracting cultures of cardiomyocytes with a high dose of TNF-α (10,000 units/ml) led to arrhythmias and complete cessation of spontaneous contractions followed by severe loss of myocyte inotropy.[63] It is known that TNF-α as well as interleukins (IL-2, IL-3, IL-6) induce the formation of stress proteins in cultured cardiomyocytes.[23,58,64] HSPs participate in cellular defense mechanisms and enable cells to survive and recover from stressful conditions.[19,24,27,28] It is believed that the heart has its own endogenous system(s) for protecting itself against ischemia–reperfusion injury and a number of HSPs that may act as chaperones in saving vital cellular proteins from degradation have been proposed.[21–28] We tested the hypothesis that TNF-α stimulates cytoprotective mechanisms in cardiomyocytes; such mechanisms can be examined by investigating the expression pattern of various stress protein genes. In an *in vitro* model based on cultured cardiomyocytes treated with TNF-α we examined gene expression of several HSPs (HSP27, HSP70 and ubiquitin).[64] TNF-α induced arrhythmia and cessation of spontaneous contractions in both a concentration- and time-dependent manner. By using molecular biological techniques we found that steady state (ubiquitin) or undetectable mRNA levels (HSP27, HSP70) were drastically increased as compared to the untreated control cells; the effects of TNF-α were maximal at 6–8 h of stimulation after which the expression of these stress genes declined. By Western blot analysis we found increased multiple bands of ubiquitin–protein conjugates in TNF-α treated cells, but no significant change in HSP27 protein accumulation was observed until 12 h. After 24 h incubation with TNF-α partial cellular necrosis was detected; hence, the induced expression of cytoprotective molecules such as stress proteins (HSP27, HSP70 and ubiquitin) in response to TNF-α may activate protective and/or repair mechanisms in cardiomyocytes which may make them more resistant toward a subsequent challenge such as ischemia. It appears that TNF-α, on the one hand, mimics cellular injury in the heart and on the other it stimulates synthesis of vital proteins like HSPs and $Mn^{2+}$ SOD making it a very interesting and relevant cytokine for the cardiovascular system. Furthermore, the ubiquitin system could play an important role in cytosolic degradation of damaged proteins in TNF-α treated cardiomyocytes where HSPs may counteract the proteolytic events and preserve many vital proteins. Thus, induction of genes conferring resistance to the cytotoxic property of TNF-α may provide a means by which cardiomyocytes can defend themselves under pathophysiological conditions.

### Interleukin-1

Interleukin-1 (IL-1) is a multifunctional cytokine, primarily involved in the regulation of inflammatory processes; it mediates most of the acute-phase response to infection including induction of fever.[65] Recent evidence indicates that IL-1 produced within tissues contributes to local inflammatory reactions.[66] Other biological activities of interleukin-1 include induction of fibroblast growth, ICAM-1 expression and growth and differentiation of B and T cells.[67] Two genes are expressed for IL-1: IL-1α and IL-1β. Although, these genes show only 20–30% amino acid homology they were shown to bind the same high-affinity receptor.[68] IL-1 does not posses a typical hydrophobic signal sequence for secretion and may be processed extracellularly by limited proteolysis from a high molecular mass intracellular precursor of 33 kDa to an active 17 kDa form.[69,70] IL-1 is produced mainly by macrophages/monocytes, T cells, B cells, fibroblasts, keratinocytes, astrocytes and endothelial cells[67] and has a wide range of target cells including cardiomyocytes and vascular smooth muscle cells. It also induces prostanoid-dependent hypotension in rabbits *in vivo*[71] and stimulates human smooth muscle cells to secrete interleukin-6.[72] In chronically ischemic myocardium where focal necrosis was documented, enhanced levels of IL-β mRNAs were found indicating a role of this cytokine in myocardial inflammation.[15] Han and co-workers[73] have observed unchanged IL-1β mRNA expression in non-failing and failing human heart by RT–PCR. IL-1β induces macrophage-type nitric oxide synthase gene expression in cardiomyocytes leading to *de novo* synthesis of NO in the heart.[74] Treatment of rat hearts with IL-1α resulted in improved ventricular systolic pressure and overexpression of $Mn^{2+}$ SOD resulting in reduced ischemia–reperfusion injury.[75]

### Interleukin-6

Interleukin-6 (IL-6), a secreted single chain protein of 28 kDa, located on chromosome 7 in human and on chromosome 5 in mouse, is another pluripotent cytokine.[67] It is released from a variety of cell types including monocytes/macrophages, fibroblasts and endothelial cells.[67,76] Recently, it was shown that IL-6 is also expressed by vascular smooth muscle cells in atherosclerotic lesions of genetically hyperlipidemic rabbits.[77] IL-6 has a wide variety of biological functions including induction of B-cell differentiation and acute-phase response.[78,79] Treatment of cultured rat vascular smooth muscle cells with recombinant IL-6 resulted in an increased c-*myc* mRNA level, followed by an increase in DNA synthesis and cell number, indicating that IL-6 may play a role in the proliferation of these cells.[80] Human vascular smooth muscle cells express and secrete IL-6 after IL-1 stimulation or during proliferation.[72] We have shown that IL-6 is expressed in the porcine heart at least at the mRNA level and its expression may be regulated by ischemia–reperfusion.[15] Furthermore, hypoxic cardiomyocytes have been shown to produce IL-6 which could contribute to ventricular dysfunction as observed after myocardial ischemia and reperfusion.[81] Hirano *et al.*[78] reported that IL-6 was expressed in cardiac myxoma cells at much higher levels than in activated lymphocytes. There is now evidence that in patients suffering from acute myocardial infarction, IL-6 may affect the progression and the healing process of this illness, because IL-6 serum levels seem to be elevated in these patients.[82] Recently, it was shown that mRNAs encoding IL-6 and ICAM-1 were induced in ischemic–reperfused myocardium and they were localized in viable myocytes adjoining the necrotic infarct suggesting a highly regulated process.[83]

### Cytokines – Mediators of Inflammation in Ischemia and Reperfusion

A variety of cytokines including IL-1, IL-6 and IL-8 as well as TNF-α have been proposed as important mediators of myocardial ischemia–reperfusion injury. For example, IL-1 was found in the circulating monocytes within hours of cardiopulmonary bypass.[84] Maximal amounts of IL-1 were observed 24 h after extracorporeal circulation. In another related study, an elevated level of plasma IL-6 was found in the patients following bypass surgery.[85] Serum levels of IL-6 have also been reported to be elevated in patients after myocardial infarction.[82] Our own laboratory has demonstrated induction of both IL-1α and IL-8 in circulating leukocytes of human patients with peak levels at 24 h post bypass.[86] Intravenous administration of IL-1 or TNF-α was associated with cardiopulmonary dysfunction in pigs subjected to cardiopulmonary bypass.[87] A monoclonal antibody to TNFα could block or attenuate the cardiopulmonary dysfunction.[88]

While a growing body of evidence suggests a role of cytokines in the inflammatory response associated with myocardial ischemia and reperfusion, the mechanism of cytokine induction remains speculative. It has been suggested that ischemia and reperfusion cause the activation of platelets and leukocytes as well as the activation of complement which results in the formation of C3a/C3a-des-Arg and C5a/C5a-des-Arg.[89] Increased levels of C3a-des-Arg and C5a-des-Arg were documented during coronary revascularization.[86] These complement-derived chemotactic factors can further activate polymorphonuclear leukocytes (PMN) as well as mononuclear phagocytes which in turn, generate excessive quantities of oxygen-derived free radicals.[90] The production of oxygen free radicals in the ischemic reperfused myocardium is well documented[91] and free radicals are known inducers of endogenous pyrogens such as IL-1.[92] The activation and accumulation of PMNs are now thought to be due to the generation of local and systemic leukocyte chemotactic factors (LCFs).[93] IL-1 was initially thought to induce chemotaxis, but is now shown not to possess any chemotactic activity; the previously reported chemotactic activity of this cytokine is believed to be due to contamination with other LCFs such as IL-8. IL-8 is one of the most powerful PMN and T-lymphocyte chemotactic factors; it can stimulate PMN adherence to endothelial cells and extracellular matrix proteins which is a manifestation of ischemic reperfusion injury.[94] IL-8 can also illicit a respiratory burst with the generation of oxygen free radicals and a rapid but transient increase in the free intracellular $Ca^{2+}$ concentration.[95] It is quite possible that the hydroxyl radical (OH·) formation associated with ischemia–reperfusion is mediated in part through IL-8 formation. Among many potential sources of *in vivo* OH· formation, PMNs represent the most significant one.[96] The involvement of PMN in the pathogenesis of myocardial ischemia reperfusion injury has been implicated in many previous studies. During phagocytosis, PMN release their granule contents with massive production of many toxic oxygen metabolites including oxygen free radicals. Very recently, phagocytosing PMNs were found to express high levels of IL-8 mRNA. There is no doubt that IL-8 represents the most powerful neutrophil chemotactic activity; it is also highly selective for PMNs. The newly expressed IL-8 can further stimulate the degranulation and respiratory burst of neutrophils, resulting in the formation of superoxide anions and hydrogen peroxide and thus potentiate a cascade of reactions leading to a gross inflammatory response.

Another mechanism by which cytokines may potentiate inflammatory response in the ischemic reperfused heart is by enhancing phospholipase $A_2$ activity. TNF-α was found to stimulate phospholipase $A_2$ and release arachidonic acid leading to the biosynthesis of cyclooxygenase products.[97] Activation of phospholipase $A_2$ in concert with the accumulation of arachidonic acid and production of prostaglandin endoperoxides and hydroperoxides are the salient features of myocardial ischemic reperfusion injury.[98] Many of the cyclooxygenase products including hydroperoxides and endoperoxides are known mediators of inflammation.

Interestingly, many of the cytokines including IL-1, IL-6 and TNF-α have been shown to stimulate nitric oxide (NO) production.[99] Reports exist to indicate that IL-1 may impair the myocardium by inhibiting intrinsic regulatory mechanisms by modulating the β-adrenergic control of cardiac L-type $Ca^{2+}$ current and that this effect is mediated by the L-arginine–nitric oxide pathway.[100] IL-1 can uncouple the β-receptor from adenylyl cyclase through a pertussis toxin-sensitive substrate such as the $G_i$ protein. The generation of NO has been suggested to play a role in the impairment of ventricular function during cytokine-mediated inflammation.[101] A number of recent studies have demonstrated a role for NO in myocardial ischemic reperfusion injury,[101,102] and there is evidence that IL-1 activates a dexamethasone-sensitive L-Arg/NO pathway.[103] The finding that cytokines induce NO production[102,103] futher supports the role for NO in the augmentation of the cytokine-mediated inflammatory response.

### Cytokines Mediate Adaptive Response in Ischemic and Reperfused Myocardium

A number of recent studies have indicated that molecular and cellular adaptation to oxidative stress makes the heart more tolerant to ischemic reperfusion injury.[104] It is generally believed that such oxidative stress adaptation is mediated by the induction of several stress proteins including antioxidants and HSPs. At low doses, cytokines such as IL-1 and TNF-α can induce the expression of HSPs by adapting the heart to oxidative stress.[59] IL-1 and IL-6 can also induce antioxidant enzymes such as $Mn^{2+}$ SOD).[105] IL-1 has been found to function as a therapeutic agent when used at low doses;[106] and it possesses the ability to downregulate its own receptors thus explaining the protective role of IL-1 to lethal challenges by hypoxia or ischemia.[107]

One of the prominant features of myocardial ischemic reperfusion injury is the development of oxidative stress. Ischemia and reperfusion potentiate the oxidative stress by at least two mechanisms. Firstly, natural antioxidative enzymes including $Mn^{2+}$ SOD, catalase, glutathione (GSH) peroxidase and GSH-reductase are reduced during ischemia and reperfusion.[108] Secondly, it is well known that oxygen-derived free radicals are produced during reperfusion of ischemic myocardium.[99] The results of our own study has demonstrated that pretreatment of heart with low doses of IL-1 can reduce oxidative stress and enhance several antioxidant enzymes as well as HSP-27 in the ischemic reperfused myocardium.[109] Such oxidative stress adaptation was associated with the reduction of ischemic reperfusion injury suggesting a strong correlation between the induction of oxidative stress inducible proteins and reduction of ischemic reperfusion injury by IL-1.

In summary, ischemia and reperfusion induce the expression of cytokines such as IL-1, IL-6, IL-8 and TNF-α. These cytokines presumably play a role in the inflammatory response associated with ischemic reperfusion injury. However, a small amount of IL-1 or TNF-α appear to induce HSPs and antioxidants that make the heart more tolerant to subsequent lethal ischemic injury. Taken together, it is tempting to speculate that ischemia–reperfusion-induced induction of cytokines reflects the adaptive response of the heart to the oxidative stress. In order to summarize

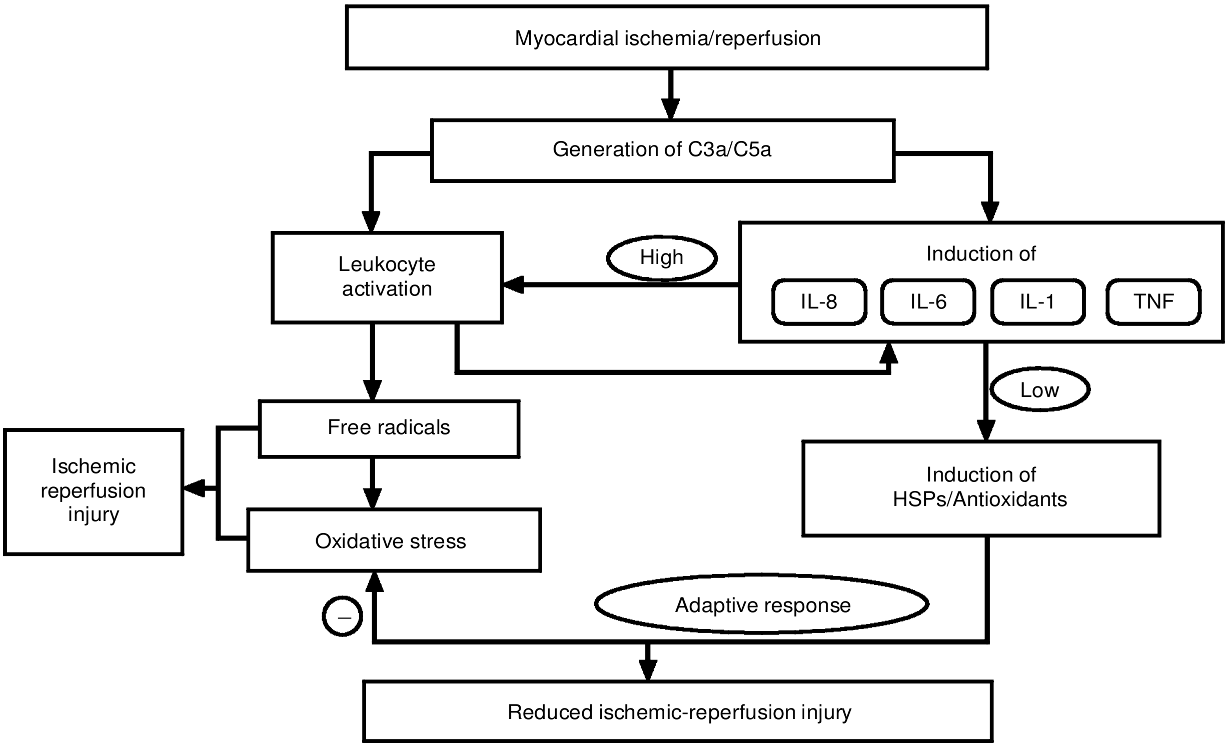

FIG. 1. A model of the role of cytokines in myocardial ischemia and reperfusion.

the existing literature in light of our own work, we propose a speculative model depicting the role of cytokines in myocardial ischemia and reperfusion (Fig. 1). We believe that in short ischemic episodes when there is a lower level of stress cytokine levels are low and the heart can enhance its own defense by inducing HSPs and antioxidants; if, however, the amount of stress is large, the induced cytokines are overwhelmed by the stress and a cascade of inflammatory reactions is initiated which leads to ischemic reperfusion injury.

### References

1. Jennings R, Schaper J, Hill M, Steenbergen C, Reimer K. Effect of reperfusion late in the phase of reversible ischemic injury. *Circ Res* 1985; **56**: 262–271.
2. Jennings RB, Sommer HM. Myocardial necrosis induced by temporary occlusion of a coronary artery in the dog. *Arch Pathol* 1960; **70**: 68–81.
3. Bolli R. Mechanisms of myocardial stunning. *Circulation* 1990; **82**: 723–738.
4. Kusuoka H, Marban E. Cellular mechanisms of myocardial stunning. *Annu Rev Physiol* 1992; **54**: 243–256.
5. Murry CE, Richard VJ, Reimer KA, Jennings RB. Ischemic preconditioning slows energy metabolism and delays ultrastructural damage during a sustained ischemic episode. *Circ Res* 1990; **66**: 913–931.
6. Schott R, Rohmann S, Braun E, Schaper W. Ischemic preconditioning reduces infarct size in swine myocardium. *Circ Res* 1990; **66**: 1133–1142.
7. Sharma HS, Wünsch M, Brand T, Verdouw PD, Schaper W. Molecular biology of the coronary vascular and myocardial responses to ischemia. *J Cardiovasc Pharmacol* 1992; **20**: S23–S31.
8. Görge G, Schmidt T, Ito BR, Pantely GA, Schaper W. Microvascular and collateral adaptation in swine hearts following progressive coronary artery stenosis. *Basic Res Cardiol* 1989; **84**: 524–535.
9. Schaper W, Schaper J. Adaptation to and defense against myocardial ischemia. *Cardiol* 1990; **77**: 367–372.
10. Andres J, Sharma HS, Knöll R, *et al.* Expression of heat shock proteins in the normal and stunned porcine myocardium. *Cardiovas Res* 1993; **27**: 1421–1429
11. Brand T, Sharma HS, Fleischmann KE, *et al.* Proto-oncogene expression in porcine myocardium subjected to ischemia and reperfusion. *Circ Res* 1992; **71**: 1351–1360.
12. Sharma HS, Maulik N, Gho BC, Das DK, Verdouw PD. Coordinated expression of heme oxygenase-1 and ubiquitin in the porcine heart subjected to ischemia and reperfusion. *Mol Cell Biochem* 1996; **157**: 111–116.
13. Frass O, Sharma HS, Fleischmann K, *et al.* Enhanced gene expression of calcium regulatory proteins in porcine stunned myocardium. *Cardiovas Res* 1993; **27**: 2037–2043.
14. Sharma HS, Verdouw PD, Lamers JMJ. Involvement of sarcoplasmic reticulum calcium pump in myocardial contractile dysfunction: Comparison between chronic pressure-overload and stunning. *Cardiovas Drugs Ther* 1994; **8**: 461–468.
15. Sharma HS, Zimmermann R. Growth Factors and development of coronary collaterals. In: Cummins P ed. *Growth Factors and the Cardiovascular System*. Kluwer Academic Publishers, 1993: 119–148.
16. Sharma HS, Tang ZH, Tang, Gho BC, Sassen LMA, Vedouw PD. Expression and immunohistochemical localization of vascular endothelial growth factor during ischemia induced ventricular dysfunction in pigs. *Circulation* 1994; **90**: 15–22.
17. Johnson PFL. Eukaryotic transcriptional regulatory proteins. *Ann Rev Biochem* 1989; **58**: 799–839.
18. Zelenka PS. Proto-oncogenes in cell differentiation. *Bio-Essays* 1990; **12**: 22–26.
19. Welch W. Mammalian stress response: cell physiology, structure/function of stress proteins, and implications for medicine and disease. *Physiological Reviews* 1992; **72**: 1063–1081.
20. Yellon D, Latchman D. Stress proteins and myocardial protection. *J Mol Cell Cardiol* 1992; **24**: 113–124.
21. Sharma HS, Snoeckx LH, Sassen LMA, *et al.* Expression and immunohistochemical localization of heat shock protein-70 in preconditioned porcine myocardium. *Annals New York Acad Sci* 1994; **723**: 491–494.
22. Sharma HS, Stahl J. Role of small heat shock proteins in the cardiovascular system. In: Knowlton AA, ed. *Heat Shock Proteins and the Cardiovascular System*. Boston: Kluwer Academic Publishers 1997.
23. Sharma HS, Stahl J, Weisensee D, Löw-Friedrich I. Cytoprotective mechanisms in cardiomyocytes. *Mol Cell Biochem* 1996; **160/161**: 217–224.
24. Ciocca DR, Oesterreich S, Chamness GC, McGuire WL, Fuqua SA. Biological and clinical implications of heat shock protein 27,000 (Hsp27): a review. *J Natl Cancer Inst* 1993; **85**: 1558–1570.
25. Knowlton AA. The role of heat shock proteins in the heart. *J Mol Cell Cardiol* 1995; **27**: 121–131.
26. Knowlton A, Brecher P, Apstein C, Ngoy S, Romo G. Rapid expression of heat shock protein in the rabbit after brief cardiac ischemia. *J Clin Invest* 1991; **87**: 139–147.
27. Heads RJ, Yellon DM, Latchman DS. Differential cytoprotection against heat stress or hypoxia following expression of specific stress protein genes in myogenic cells. *J Mol Cell Cardiol* 1995; **27**: 1669–1678.
28. Mestril R, Dillmann WH. Heat shock proteins and protection against myocardial ischemia. *J Mol Cell Cardiol* 1995; **27**: 45–52.
29. Hayashi T, Takada K, Matsuda M. Post-transient ischemia increase in ubiquitin conjugates in the early reperfusion. *NeuroReport* 1992; **3**: 519–520.
30. Hershko A, Ciechanover A. The ubiquitin system for protein degradation. *Annu Rev Biochem* 1992; **61**: 761–807.
31. Jentsch S. Ubiquitin-dependent protein degradation: a cellular perspective. *Trends in Cell Biology* 1992; **2**: 98–103.
32. Das DK, Engelman RM, Kimura Y. Molecular adaptation of cellular defences following preconditioning of the heart by repeated ischemia. *Cardiovas Res* 1993; **27**: 578–584.
33. Maulik N, Sharma HS, Das DK. Induction of heme oxygenage gene expression during the reperfusion of ischemic rat myocardium. *J Mol Cell Cardiol* 1996; **28**: 1261–1270.
34. Thompson NL, Basoberry F, Spier EH, *et al.* TGF-β1 in acute myocardial infarction in rats. *Growth Factor* 1988; **1**: 91–99.
35. Eghbali M. Cellular origin and distribution of transforming growth factor β in the normal rat myocardium. *Cell Tissue Res* 1989; **256**: 553–558.
36. Wünsch M, Sharma HS, Markert T, *et al.* In situ localization of transforming growth factor-β1 in porcine heart: Enhanced expression after chronic coronary artery constriction *J Mol Cell Cardiol* 1991; **23**: 1051–1062.
37. Roberts AB, Sporn MB, Assoian, RK, *et al.* TGF-β: Rapid induction of fibrosis and angiogenesis in vivo and stimulation of collagen formation in vitro. *Proc Natl Acad Sci (USA)* 1986; **83**: 4167–4171.
38. Mustoe TA, Pierce GF, Thomason A, Gramates P, Sporn MB, Deuel TF. Accelerated healing of incisional wounds in rats induced by transforming growth factor-beta. *Science* 1987; **237**: 1333–1336.
39. Folkman J, Shing Y. Angiogenesis. *J Biol Chem* 1992; **267**: 10931–10934.
40. Lefer AM. Mechanism of the protective effects of transforming growth factor-β in reperfusion injury. *Biochem Pharmacol* 1991; **42**: 1323–1327.
41. Lefer AM, Tsao P, Aoki N, Palladino MJ. Mediation of cardioprotection by transforming growth factor-β. *Science* 1990; **249**: 61–64.
42. MacLellan WR, Brand T, Schneider MD. Transforming growth factor-beta in cardiac ontogeny and adaptation. *Circ Res* 1993; **73**: 783–791.
43. Roberts AB, Sporn MB. Physiological actions and clinical applications of transforming growth factor-beta (TGF-beta). *Growth Factors* 1993; **8**: 1–9.
44. Tracey KJ, Cerami A. Tumor necrosis factor: A pleiotropic cytokine and therapeutic target. *Ann Rev Med* 1994; **45**: 491–503.
45. Sherry B, Cerami A. Cachetin/tumor necrosis factor exerts endocrine, paracrine, and autocrine control of inflammatory responses. *J Cell Biol* 1988; **107**: 1269–1277.
46. Ikeda U, Ikeda M, Kano S, Shimada K. Neutrophil adherence to rat cardiac myocyte by proinflammatory cytokines. *J Cardiovas Pharmacol* 1994; **23**: 647–652.
47. Sharma HS, Weisensee D, Löw-Friedrich I, Schoeppe W, Schaper W. Vascular endothelial growth factor expression in cardiac myocytes in vitro and its upregulation by tumor necrosis factor-α. *J Cell Biochem* 1993; **17D**: 216.
48. Tartaglia LA, Weber RF, Figari IS, Reynolds C, Palladino MJ, Goeddel DV. The two different receptors for tumor necrosis factor mediate distinct cellular responses. *Proc Natl Acad Sci (USA)* 1991; **88**: 9292–9296.
49. Wiegmann K, Schutze S, Kampen E, Himmler A, Machleidt T, Kronke M. Human 55-kDa receptor for tumor necrosis factor coupled to signal transduction cascades. *J Biol Chem* 1992; **267**: 17997–18001.
50. Heller RA, Kronke M. Tumor necrosis factor receptor-mediated signaling pathways. *J Cell Biol* 1994; **126**: 5–12.
51. Rothe J, Gehr G, Loetscher H, Lesslauer W. Tumor necrosis factor receptors– structure and function. *Immunol Res* 1992; **11**: 81–90.
52. Vaddi K, Nicolini FA, Mehta P, Mehta JL. Increased secretion of tumor necrosis factor-alpha and interferon-gamma by mononuclear leukocytes in patients with ischemic heart disease. Relevance in superoxide anion generation. *Circulation* 1994; **90**: 694–699.
53. Torre AG, Kapadia S, Lee J, *et al.* Tumor necrosis factor-alpha and tumor necrosis factor receptors in the failing human heart. *Circulation* 1996; **93**: 704–711.
54. Herskowitz A, Choi S, Ansari AA, Wesselingh S. Cytokine mRNA expression in postischemic/reperfused myocardium. *Am J Pathol* 1995; **146**: 419–428.
55. Maury CP, Tempo AM. Circulating tumor necrosis factor-α (cachectin) in myocardial infarction. *Am J Pathol* 1989; **139**: 709–715.
56. Eddy LJ, Goeddel DV, Wong GH. Tumor necrosis factor-α pretreatment is protective in a rat model of myocardial ischemia-reperfusion injury. *Biochem Biophys Res Comm* 1992; **184**: 1056–1059.
57. Yokoyama T, Vaca L, Rossen RD, Durante W, Hazarika P, Mann DL. Cellular basis for the negative inotropic effects of tumor necrosis factor-alpha in the adult mammalian heart. *J Clin Invest* 1993; **92**: 2303–2312.
58. Low FI, Weisensee D, Mitrou P, Schoeppe W. Cytokines induce stress protein formation in cultured cardiac myocytes. *Basic Res Cardiol* 1992; **87**: 12–18.
59. Kaur P, Welch WJ, Saklatvala J. Interleukin 1 and tumour necrosis factor increase phosphorylation of the small heat shock protein. Effects in firbroblasts, Hep G2 and U937 cells. *FEBS Lett* 1989; **258**: 269–273.
60. Mehlen P, Mehlen A, Guillet D, Preville X, Arrigo A-P. Tumor necrosis factor-α induces changes in the phosphorylation, cellular localization, and oligomerization of human hsp27, a stress protein that confers cellular resistance to this cytokine. *J Cell Biochem* 1995; **58**: 248–259.
61. Reithmann C, Gierschik P, Werdan K Jakobs KH: Tumor necrosis factor-α up-regulates $G_I$ alpha and G beta proteins and adenylyl cyclase responsiveness in rat cardiomyocytes. *Eur J Pharmacol* 1991; **206**: 53–60.
62. Engel K, Ahlers A, Brach MA, Herrmann F, Gaestel M. MAPKAP Kinase 2 is activated by heat shock and TNF-α: In vivo phosphorylation of small heat shock protein results from stimulation of the MAP Kinase cascade. *J Cell Biochem* 1995; **57**: 321–330.
63. Weisensee D, Bereiter HJ, Schoeppe W, Low FI. Effects of cytokines on the contractility of cultured cardiac myocytes. *Int J Immunopharmacol* 1993; **15**: 581–587.
64. Sharma HS, Weisensee D, Low FI. Tumor necrosis factor-alpha-induced cytoprotective mechanisms in cardiomyocytes. Analysis by mRNA phenotyping. *Ann N Y Acad Sci* 1996; **793**: 267–281.
65. Lord PCW, Wilmoth LMG, Mizel SB, McCall CE. Expression of interleukin-1α and β genes by human blood polymorphonuclear leukocytes. *J Clin Invest* 1991; **87**: 1312–1321.
66. Dinarello CA. Biology of interleukin 1. *FASEB J* 1988; **2**: 108–115.
67. Akira S, Hirano T, Taga T, Kishimoto T. Biology of multifunctional cytokines: IL 6 and related molecules (IL 1 and TNF). *FASEB J* 1990; **4**: 2860–2867.
68. Kilian PL, Kaffka KL, Stern AS, *et al.* Interleukin-1α and interleukin-1β bind to the same receptor on T cells. *J Immunol* 1986; **136**: 4509–4514.
69. Whicher JT, Evans SW. Cytokines in disease. *Clin Chem* 1990; **36/7**: 1269–1281.
70. Rubartelli A, Cozzolino F, Talio M, Sitia R. A novel secretory pathway for interleukin-1β, a protein lacking a signal sequence. *EMBO J* 1990; **9**: 1503–1510.
71. Okusawa S, Gelfand JA, Ikejima T, Connoly RJ, Dinarello CA. Interleukin 1 induces a shock-like state in rabbits. Synergism with tumor necrosis factor and the effect of cyclooxygenase inhibition. *J Clin Invest* 1988; **81**: 1162–1172.
72. Loppnow H, Libby P. Proliferating interleukin 1 activated human vascular smooth muscle cells secrete copius interleukin 6. *J Clin Invest* 1990; **85**: 731–738.
73. Han RO, Ray PE, Baughman KL, Feldman AM. Detection of interleukin and interleukin-receptor mRNA in human heart by polymerase chain reaction. *Biochem Biophys Res Comm* 1991; **181**: 520–523.
74. Tsujino M, Hirata Y, Imai T, *et al.* Induction of nitric oxide synthase gene by interleukin-1β in cultured rat cardiomyocytes. *Circulation* 1994; **90**: 375–383.
75. Nogae C, Makino N, Hata T, *et al.* Interleukin 1 alpha-induced expression of manganous superoxide dismutase reduces myocardial reperfusion injury in the rat. *J Mol Cell Cardiol* 1995; **27**: 2091–2099.
76. Bendtzen K. Interleukin 1, interleukin 6 and tumor necrosis factor in infection, inflammation and immunity. *Immunol Lett* 1988; **19**: 183–192.
77. Hennein HA, Ebba H, Rodriguez JL, *et al.* Relationship of the proinflammatory cytokines to myocardial ischemia and dysfunction after uncomplicated coronary revascularization. *J Thorac Cardiovasc Surg* 1994; **108**: 626–635.
78. Hirano T, Yasukawa K, Harada H, *et al.* Complementary DNA for a novel human interleukin (BSF-2) that induces B lymphocytes to produce immunoglobulin. *Nature* 1986; **324**: 73–76.
79. Geiger T, Andus T, Klapproth J, Hirano T, Kishimoto T, Heinrich PC. Induction of acute phase-proteins by interleukin 6 in vivo. *Eur J Immunol* 1988; **18**: 717–721.
80. Morimoto S, Nabata T, Koh E, *et al.* Interleukin-6 stimulates proliferation of cultured vascular smooth muscle cells independently of interleukin-1β. *J Cardiovas Pharmacol* 1991; **17** (suppl. 2): S117–S118.
81. Yamauchi TK, Ihara Y, Ogata A, Yoshizaki K, Azuma J, Kishimoto T. Hypoxic stress induces cardiac myocyte-derived interleukin-6. *Circulation* 1995; **91**: 1520–1524.
82. Ikeda U, Ohkawa F, Seino Y, *et al.* Serum interleukin 6 levels become elevated in acute myocardial infarction. *J Mol Cell Cardiol* 1992; **24**: 579–584.
83. Kukielka GL, Youker KA, Hawkins HK, *et al.* Regulation of ICAM-1 and IL-6 in myocardial ischemia: effect of reperfusion. *Ann N Y Acad Sci* 1994; **723**: 258–270.
84. Haeffner-Cavaillon N, Roussellier N, Ponzio O, *et al.* Induction of interleukin-1 production in patients undergoing cardiopulmonary bypass. *J Thorac Cardiopulmo Surg* 1989; **98**: 1100–1106.
85. Finkel MS, Hoffman RA, Shen L, Oddis CV, Simmons RL, Hattler BG. Interleukin-6 as a mediator of stunned myocardium. *Am J Cardiol* 1993; **71**: 1231–1232.
86. Kalfin RE, Engelman RM, Rousou JA, *et al.* Induction of interleukin-8 expression during cardiopulmonary bypass. *Circulation* 1993; **88**: 401–406.
87. Kruse-Elliott KT, Olson NC. CGS 8515 and indomethacin attenuate cytokine-induced cardiopulmonary dysfunction in pigs. *Am J Physiol* 1993; **264**: H1075–H1086.
88. Walsh CJ, Sugerman HJ, Mullen PG, *et al.* Monoclonal antibody to tumor necrosis factor α attenuates cardiopulmonary dysfunction in porcine Gram-negative sepsis. *Arch Surg* 1992; **127**: 138–145.
89. Fosse E, Mollnes TE, Ingvaldsen B. Complement activation during major operations with or without cardiopulmonary bypass. *J Thoracic Cardiovasc Surg* 1987; **93**: 860–866.
90. Hind CRK, Griffin JF, Pack F, *et al.* Effect of cardiopulmonary bypass on circulating concentrations of leukocyte elastase and free radical activity. *Cardiovasc Res* 1988; **22**: 37–41.
91. Das DK, Engelman RM. Mechanisms of free radical generation in ischemic and reperfused myocardium In: Das DK, Essman WB eds. *Oxygen Radicals: Systemic Events and Disease Processes*. Basel: Krager, 1990: 97–128.
92. Chenoweth DE, Cooper SW, Hugli TE, Stewart RW, Blackstone EH, Kirklin JW. Complement activation during cardiopulmonary bypass. *N Engl J Med* 1981; **304**: 497–503.
93. Nilsson L, Brunnkvist S, Nilsson U, *et al.* Activation of inflammatory systems during cardiopulmonay bypass. *Scand J Thorac Cardiovasc Surg* 1988; **22**: 51–53.
94. Lucchesi BR, Mullane KM. Leukocytes and ischemia induced myocardial injury. *Ann Rev Pharmacol Toxicol* 1986; **26**: 201–224.
95. Thelen M, Peveri P, Kernen P, Tsharner VV, Walz A, Baggiolini M. Mechanism of neutrophil activation by NAF, a novel monocyte-derived peptide agonist. *FASEB J* 1988; **2**: 2702–2706.
96. Bagchi D, Das DK, Engelman RM, Prasad MR, Subramanian R. Polymorphonuclear leucocytes as potential source of free radicals in the ischemic-reperfused myocardium. *Eur Heart J* 1990; **11**: 800–813.
97. Spriggs DR, Sherman ML, Imamura K, *et al.* Phospholipase $A_2$ activation and autoinduction of tumor necrosis factor gene expression by tumor necrosis factor. *Cancer Res* 1990; **50**: 7101–7107.
98. Otani H, Engelman RM, Rousou JA, Breyer RH. Enhanced prostaglandin synthesis due to phospholipase breakdown in ischemic-reperfused myocardium. Control of its production by a phospholipase inhibitor or free radical scavengers. *J Mol Cell Cardiol* 1986; **18**: 953–961.
99. Balligand JL, Ungureanu LD, Simmons WW, *et al.* Cytokine-inducible nitric oxide synthase (iNOS) expression in cardiac myocytes. Characterization and regulation of iNOS expression and detection of iNOS activity in single cardiac myocytes *in vitro*. *J Biol Chem* 1994; **269**: 27580–27588.
100. Rozanski GJ, Witt RC. IL-1 inhibits β-adrenergic control of cardiac calcium current: role of L-arginine/nitric oxide pathway. *Am J Physiol* 1994; **267**: H1753–H1758.
101. Engelman DT, Watanabe M, Engelman RM, *et al.* Constitutive nitric oxide release is impaired after ischemia and reperfusion. *J Thorac Cardiovasc Surg* 1995; **110**: 1047–1053.
102. Evans HG, Lewis MJ, Shah AM. Interleukin 1β modulates myocardial contraction via dexamethasone sensitive production of nitric oxide. *Cardiovasc Res* 1993; **27**: 1486–1490.
103. Schulz R, Nava E, Moncada S. Induction and potential biological relevance of a $Ca^{2+}$-independent nitric oxide synthase in the myocardium. *Br J Pharmacol* 1992; **105**: 575–580.
104. Maulik N, Watanabe M, Engelman DT, Engelman RM, Das DK. Oxidative stress adaptation improves postischemic ventricular recovery. *Mol Cell Biochem* 1995; **144**: 67–74.
105. Ono M, Kohda H, Kawaguchi T, *et al.* Induction of Mn-superoxide dismutase by tumor necrosis factor, interleukin-1 and interleukin-6 in human hepatoma cells. *Biochem Biophys Res Commun* 1992; **182**: 1110–1107.
106. Yamada M, Sohmura Y, Nakamura S, Hashimoto M. Interleukin-1α: its possible roles in cancer tharapy. *Biotherapy* 1989; **1**: 327–338.
107. Matsushima K, Yodoi J, Tagaya Y, Oppenheim JJ. Down regulation of interleukin-1 receptor expression by IL-1 and fate of internalized $^{125}$I-

labeled IL-1α in a human large granular lymphocyte cell line. *J Immunol* 1986; **137**: 3183–3188.
108. Das DK, Engelman RM, Rousou JA, Breyer RH, Otani H, Lemeshow S. Pathophysiology of superoxide radical as potential mediator of reperfusion injury in pig heart. *Basic Res Cardiol* 1986; **81**: 155–166.
109. Maulik N, Engelman RM, Wei Z, Lu D, Rousou JA, Das DK. Interleukin-α preconditioning reduces myocardial ischemia reperfusion injury. *Circulation* 1993; **88**: 387–394.

**Received 16 May 1997**
**revised and accepted 27 May 1997**
